# Closed Systems for Long-Term Propagation of the Marine Tunicate *Botryllus schlosseri* Isolated from Natural Seawater

**DOI:** 10.3390/life16071102

**Published:** 2026-07-01

**Authors:** Jens Hamar, Weizhen Dong, Brenda Luu, Mandy Lin, Isabel Enriquez, Maxime Leprêtre, Alison M. Gardell, Baruch Rinkevich, Dietmar Kültz

**Affiliations:** 1 Department of Animal Sci. & Genome Center, University of California Davis, Meyer Hall, One Shields Ave., Davis, CA 95616, USA; jchamar@ucdavis.edu (J.H.); vdong@ucdavis.edu (W.D.); bpluu@ucdavis.edu (B.L.); maylin@ucdavis.edu (M.L.); inenriquez@ucdavis.edu (I.E.); mlepretre@ucdavis.edu (M.L.); 2 School of Interdisciplinary Arts and Sciences, University of Washington Tacoma, 1900 Commerce St., Tacoma, WA 98402, USA; agardell@uw.edu; 3 Israel Oceanography & Limnological Research, National Institute of Oceanography, Haifa 3109701, Israel; buki@ocean.org.il

**Keywords:** live food, tunicates, recirculating aquaculture system (RAS), microalgae, zooid

## Abstract

Advanced methodologies for *Botryllus schlosseri* artificial seawater systems are needed to decrease dependency of large-scale culture on natural seawater and expand this important new model organism to more inland laboratories. We constructed two botryllid tunicate customized closed aquaculture systems, a static system consisting of aerated jars fed commercial filter feeder diet, and a recirculating aquaculture system (RAS) consisting of pertinent marine RAS components fed live microalgae and zooplankton diets. Initially, static tunicate culture yielded exponential growth in contrast to poor survival and negligible growth observed in RAS tunicates. RAS modifications were made to increase water treatment proficiency, which improved tunicate survival and growth. Experiments were performed isolating feed and water type as variables differentiating static and RAS and evaluating their specific effects. Live feed promoted five-fold greater growth relative to a commercial concentrate diet. Tunicates maintained in optimized RAS water achieved two-fold faster growth relative to animals in freshly prepared artificial seawater. Subsequent procedural modifications combined with the RAS revisions resulted in growth rates comparable to the static system. Both optimized systems are suitable for long-term husbandry of botryllid tunicate populations supporting both sexual and asexual modes of reproduction, with a current RAS residence time of over 24 months.

## 1. Introduction

Many of the scientific advances relevant to human health and nutrition were initiated in simplified non-human organisms. Notable examples include: the discovery of gene arrangement and genetic inheritance through chromosomes using *Drosophila* [1], the molecular basis of vertebrate development using zebrafish [2], and the initial characterization of human cyclins in yeast [3]. Scientific advances in mechanistic characterization of biological processes, such as understanding immune recognition pathways and tissue or organ regeneration processes, require further development of suitable model organisms to reveal better insight into the underlying mechanisms. The emergence of tunicates as invertebrate models of vertebrate physiology is supported by their status as an ideal clade of organisms that is closely related to vertebrates but has many unique physiological abilities (e.g., whole body regeneration). Many aquatic invertebrates possess favorable attributes as models, such as simplified physiology, transparent anatomy, rapid clonal propagation capability, and flexible reproduction strategies. Tunicates combine these benefits with high relevance to vertebrate physiology due to their close phylogenetic proximity as a chordate [4]. More specifically, the colonial tunicate *B. schlosseri* has already demonstrated high value as a model for many important biological processes, including allorecognition [5], aging, stem cell biology [6], regeneration [7], and ecotoxicity [8]. However, a key bottleneck for developing this species as an emerging model organism is the lack of a culture system that is self-contained and easy to manage in any location. Routine use of these animals in scientific studies requires immediate access to wild populations or, in most cases, clean and genetically defined strains of laboratory populations that can be easily propagated under reproducible, stable conditions. Tunicate propagation is typically done using seawater directly sourced from coastal waters. Methods of static *B. schlosseri* aquaculture have been established involving regular water changes with natural seawater [9], but practical application of these approaches requires marine laboratories with convenient coastal access. Advancing knowledge and expertise in proficient aquaculture of these animals using recirculating artificial seawater systems would make this powerful new model accessible to a vastly larger range of laboratories.

An alternative to naturally sourced seawater is to prepare it from dehydrated salt mixes. Such synthetic seawater is commonly used to maintain marine animals in closed systems. However, in flow-through or direct replacement systems, synthetic seawater is expensive, ecologically unsustainable, and impractical on a large scale [10,11]. Recirculating aquaculture systems (RAS) have been identified as a means to minimize cost by reducing the use of consumable resources (i.e., water and sea salt) through conditioning and reuse of water [12]. They can create artificial marine environments that are ecologically sustainable and suitable for the long-term culture of marine species in areas where natural seawater is unavailable or its use is impractical. Additional benefits include better control over environmental/abiotic parameters [13], conservation of invested cost into modification of the source water (such as temperature change or salinity adjustment), increased biosecurity through protection from potentially harmful impacts via the source water (i.e., introduction of toxins and pathogens), and reduction in waste effluent to the environment.

In many areas, recirculating aquaculture systems (RAS) have made aquatic culture possible, or at least more cost-effective, where suitable water sources are limited [14] and allowed for the inland culture of diverse phyla of marine species, including fish (i.e., clownfish [15,16], pompano [17]) and invertebrates (crustaceans, echinoderms [18], molluscs [19], and corals [20]) in inland areas without reliance on natural seawater. A proficient RAS has recently been established for another colonial tunicate (*Botrylloides diegensis*) [21]. The objective of this project is to build on this prior advancement and eliminate reliance on natural seawater for the culture of *B. schlosseri* by constructing artificial seawater systems (both static and RAS) that are optimized for generating a steady supply of healthy, actively growing animals that can robustly support scientific research using this intriguing model organism.

## 2. Materials and Methods

### 2.1. Animal Collection

Parent stock animals were collected from Northern California floating docks within Berkely Marina (BM), Bodega Bay (BB), and San Francisco Harbor (SFH). Adult wild *B. schlosseri* and *Botrylloides* spp. were collected and held jointly in the initial RAS only after the first field collection. Wild-caught animals from all other field collections were brought into the laboratory and held in isolation tanks for the collection of *B. schlosseri* larvae. Larvae were collected onto 2” × 3” glass slides (#6686K20, Thomas Scientific, Swedesboro, NJ, USA) from aggregates of adult animals approximately 1 inch in diameter using two methods: (1) By tying colonies to slides and placing them across from a clean slide in a holder; and (2) By placing the aggregate within a box with glass slides lining the interior. These collections typically consisted of five to ten adult aggregates, which would yield several hundred to a thousand larvae colonized onto 10 to 50 glass slides. Clean slides colonized by the free-swimming larval stage (1 to 3 days post-collection of wild colonies) were removed and transferred to animal housing units (AHU). Slides were examined using a EZ4W microscope (Leica, Wetzlar, Germany) every 3 to 5 days. Colonies showing heavy contamination (visible parasites within the tissue, algae and other micro-organisms growing on or beneath the tunic), poor health (discolored or necrotic tunic, retracted and compact ampullae, darkened color) or lack of growth were culled from the slide. To decrease the density of slides containing multiple healthy colonies, single colonies of 4 to 6 zooids were transferred to new slides. As previously described [22], transfer was performed by scraping colonies off their current slide with a razor blade firmly pressed against the slide at a 45-degree angle, gently sliding the colony onto a clean slide with a paint brush, followed by incubation in a humidity chamber for 30–60 min before submerging into their designated AHU.

### 2.2. Animal Housing

Long-term growth and propagation of selected colonies were achieved in AHUs specific to the culture system. AHUs of the static system consisted of lightly aerated 2.8 L static glass jars. AHUs of the RAS consisted of one 115 L glass aquarium and flow-through clear plastic zebrafish tanks from Aquaneering Inc. (San Diego, CA, USA): four 9.5 L tanks (cat# ZT950), and fourteen 1.8 L tanks (cat# ZT180). Static system slides were held in glass slide holders. To reduce algae growth underneath the tunic caused by light penetrating the back side of slides, the RAS slides were held in custom-manufactured slide holders with opaque black acrylic backing. Ten slide capacity holders were used for the 9.5 L AHUs and a single-slide capacity holder for the 1.8 L AHUs.

### 2.3. Feeding and Maintenance

Initially, tunicates in both systems were fed a mixture of Roti-Rich (Florida Aqua Farms Inc., Dade City, FL, USA) and Phyto-Feast Live (Reed Mariculture Inc., Campbell, CA, USA). RAS tunicates were transitioned to a live feed mix consisting of four microalgae species (*Tetraselmis* sp., *Nannochloropsis* sp., *Dunaliella salina*, and *Isochrysis galbana*) and marine rotifers (*Brachionus plicatilis*). Animals cultured in the Static system were fed twice per week with 100 µL Roti-Rich and 100 µL Phyto-Feast. RAS tunicates were initially fed every other day. For the RAS, 200 mL of each microalgae culture was mixed with 800 mL rotifer culture and enough seawater to bring the total volume to 2 L, which was divided among the AHUs. Flow to AHUs was turned off for 1–2 h during maintenance to increase retention time with feed. The revised RAS feeding frequency was eventually increased to daily to increase growth rates. For both systems, stock artificial seawater (AS) was prepared by mixing a dry artificial seawater mix (Red Sea Coral Pro Salt, product# R11065, Red Sea Fish Pharmacy, Houston, TX, USA) with deionized, dechlorinated facility water in a 150 L reservoir. For the static system, 100% water changes were performed using 30 ppt AS once per week on each AHU. For the RAS, 24 L of water was siphoned from the refugium (RFG) and replaced with fresh AS. Salinity and temperature for all systems were checked daily and maintained at 30 ppt and between 19 and 20 °C, respectively. Colonies were cleaned weekly using soft brushes. Debris surrounding the tunicate colonies was carefully removed by scraping the glass slides, taking care not to damage the ampullae. Ammonia, nitrite, and nitrate were checked weekly using API test kits (product numbers LR8600, 26, and LR1800, respectively, MARS Fishcare, Chalfont, PA, USA) for the initial RAS, but were reduced to intermittent after it was determined that the RAS capacity and water turnover rate relative to the inhabitant biomass produced an environment that never reached the minimum detection limits of the test kits. The detection limits are 0.25 mg/L for the ammonia and nitrite test kits and 5 mg/L for the nitrate kit.

### 2.4. Live Feed Culture

All microalgae strains were cultured in 2 L glass jars with a flat glass top and a bottom drain with a dispensing valve. Vessels were exposed to continuous lighting using a 37-watt LED light (StingRAY 2–48” LED Aquarium Light, model#LT-FX-FL48, Finnex, Chicago, IL, USA) and lightly aerated with air filtered through a 0.22 micron syringe filter (#SLCPR33RB, Millipore Express, Burlington, MA, USA). Each vessel received 1 L of sterile 33 ppt AS supplemented with 200 µL part A and 200 µL part B of Fritz Aquatics F2 media per L AS twice per week. Rotifers were maintained in an aerated plastic 8 L conical bottom container with daily 10% water changes and feeding of resuspended dry yeast powder (*Saccharomyces cerevisiae*).

### 2.5. Initial RAS

The RAS was constructed using a 124 cm H × 168 cm L × 56 cm W stainless rack as the frame and including the following standard marine husbandry components: a 30 cm H × 132 cm L × 79 cm W, 150 L plastic stock tank (Rubbermaid, Wooster, OH, USA, mfg #424300BLA) as the Sump (SMP); circulating water pump (PMP), 20 gpm submersible pump (Danner model#12B); biofilter (BFR) consisting of a 89 cm H × 58 cm diameter 210 L polyethylene drum (Uline, Pleasant Prairie, WI, USA) filled with 30 L of Bulk Reef Supply (Golden Valley, MN, USA) 3.8 cm diameter plastic media bioballs (product# 000641); a 20” mechanical filter (MFR) containing a 200 µM cartridge filter (H2Only, Stonypoint, NY, USA, part#200M-WP-20); two temperature control chillers (CHL), AquaEuro USA (San Pedro, CA, USA) Max Chill 1/13 HP chiller (model# MC-1/13HP); refugium (RFG) contained in a 30 cm H × 69 cm L × 51 cm W 115 L glass tank illuminated by a 26 watt full spectrum LED light (Tunze, Penzberg, Germany, cat #8850); foam fractionator (FFR), Bubble Magus Curve 9 (model# BM-CURVE 9, Rancho Cucamonga, CA, USA); and a 40 watt UV sterilizer (UVS), IceCap (Slidell, LA, USA, product# IC-HOUV-40W). Pressurized water lines from the PMP and gravity feed return lines were constructed from 3/4” and 1.5” schedule 40 PVC pipe, respectively.

### 2.6. Static System

The static system was modeled after that previously described in Rinkevich and Shapira [22]. Larvae of wild colonies were spawned and attached to glass slides at the UC Davis Cole B facility within one week after field collection. These lab-born colonies developed from oozooids and were maintained vertically on glass slides in 2.8 L glass tanks containing 30 ppt standing AS at a constant temperature of 20 °C and aerated by air stones as previously described [22]. All genotypes used in this study were reared in individual tanks to avoid competition [23]. AS for each tank was changed every week, and colonies were gently cleaned once a week using soft brushes. All experimental colonies were in good health and had been born and reared in stable lab conditions for at least 3 months.

### 2.7. Revised RAS

The revised RAS includes all the components from the initial system with the following modifications: Total system circulation was divided into two water pumps (PMP), PMP1 = 20 gpm submersible pump (Danner model#12B) and PMP2 = variable speed DC submersible pump (Simplicity Aquatics, Nashville, TN, USA, 2100DC Pump, model# SIM2005); a 38 L foam fractionator tank (FFT) to contain the FFR, 10 Gallon Polyethylene Tamco^®^ Tank-13” Dia. × 19” Hgt. (item # 3005); and a 64 L polyethylene mixing tank (MXT) to contain PMP2, 17 Gallon Polyethylene Tamco^®^ Tank-18” Dia. × 15” Hgt. (item # 3010, U.S. Plastic Corp., Lima, OH, USA).

### 2.8. Growth Quantification

Growth data were collected for individual genotypes defined as all asexually produced progeny from a single sexually produced oozooid. For each recorded genotype, the numbers of individual animal units of a colony (zooids) were periodically recorded. The number of systems (a system being a discrete cluster of zooids averaging 8–12 zooids) was recorded as the unit of growth in the revised RAS during a period of 3 weeks in 2025, when no animals were harvested for experiments to avoid under-representation of growth through artificial decreases in colonies. Growth rate in the static system was estimated from genotypes with complete measurements from day 1 through day 42 (genotypes S01, S03, S09, S10, S11, S12, S13, S14, and S15). Using the lm() function in R, exponential growth was estimated using ordinary least squares linear regression of ln-transformed zooid number versus time (days).

### 2.9. Feeding Experiment

Two slides per genotype (average of 3 systems, 30 zooids per slide) from three static system-raised tunicate genotypes were selected for exposure to two feeding treatments: the commercial feeding regime (CF) described above for the static culture system and the live feed regime (LF) used in the RAS. Each treatment group received one slide from each genotype for a total of six replicates per treatment. All replicates were cultured as described above using the static system in 2.8 L jars with light aeration. Protein concentration (quantified by BCA assay) was used to equilibrate feed amount between the two treatment groups at a calculated 4 mg protein per dose (2.5 mg from RotiRich + 1.5 mg from Phyto-Feast for the CF group and 2.7 mg from rotifer culture + 1.3 mg from microalgae mix for the LF group). For the CF group, 1 mL of Roti-Rich and 1 mL of Phyto-Feast were mixed into 10 mL of water, from which 1 mL was distributed to each replicate. For the LF group, 57 mL of each microalgae culture was mixed with 225 mL of rotifer culture, from which 150 mL was distributed to each replicate. Zooid counts and imaging were performed on each replicate at weekly intervals for 3 weeks. For each treatment group, the mean relative change in zooid number (RCZN) was calculated as the total number of zooids at the final time point divided by the number of zooids at the start of the experiment for each genotype.

### 2.10. Water Experiment

Ten individual genotype RAS-raised slides of tunicates (one 6–7 zooid system/slide) were randomly allocated to two treatment groups (5 replicates/group): one receiving only freshly prepared AS and the other receiving water from loop 1 of the revised RAS. With the exception of the water source for water changes, all replicates were cultured as described above using the static system in 700 mL glass jars with light aeration. Imaging and zooid counts were performed on each replicate twice per week for 3 weeks. For each treatment group, RCZN was calculated as described above.

### 2.11. Statistics

All statistical analyses were performed using Rstudio version 2021.09.1. One-tailed Welch and two-sample t tests were performed on all RCZN comparisons. Normality of data was confirmed by the Shapiro–Wilk test. Unequal variance between groups was determined by the F-test.

## 3. Results

### 3.1. Initial RAS

**Figure 1:**
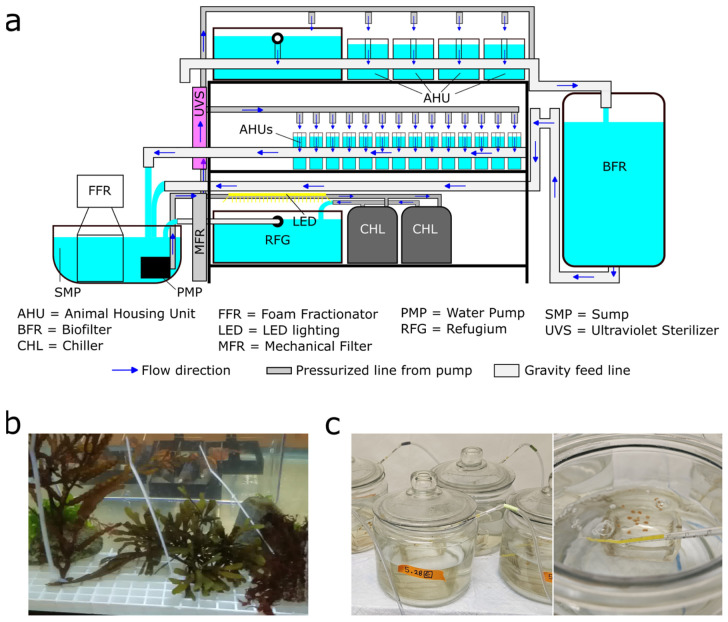
Initial culture systems. Schematic of initial RAS with flow directionality (**a**) and macrophyte population of the refugium (**b**). Images of static system AHUs (**c**).

The completed 660 L total volume initial RAS (Figure 1a) was arranged with three elevated levels on the stainless steel rack: the top level supported 5 AHUs (one 115 L and four 10 L); the second level supported 14 1.8 L AHUs; and the bottom level supported the 115 L refugium and two CHLs. The SMP and BF were located at opposite ends of the rack. The SMP was at the lowest point to which all water from the system flowed back by gravity. It contained the single circulation PMP and the FF. Pressure-driven water from the PMP was split into two paths. One path directed flow through the MFR, then through the CHLs, and then to the RFG, which gravity fed back to the SMP. The second path directed flow through the UVS, and to two distribution manifolds feeding the top and second-level AHUs. Effluent water from the top-level AHUs is gravity-fed into the BF and then back to the SMP. Effluent from the second-level AHUs is gravity-fed directly back to the SMP. The refugium was illuminated with the LED light mounted to the bottom of the second level and populated with five different species of macrophytes (macrophyte fragments or attached to small rocks) collected at the BB collection site (Figure 1b). In combination, the water turnover rate and assimilation of nitrogenous wastes by refugium macrophytes were sufficient to keep system ammonia, nitrite, and nitrate below detectable limits at all times.

### 3.2. Static System

The completed static system consisted of 20 individual AHUs (Figure 1c), each containing a single *B. schlosseri* genotype divided among three levels of a 142 cm H × 137 cm L × 33 cm W stainless steel rack. The single aerator distributed air to each AHU through individual lines connected to a filtered 1 mL serological pipette. The water turnover rate was sufficient to keep ammonia levels below detectable limits.

### 3.3. Growth Rates in Initial Aquaculture Systems

**Figure 2:**
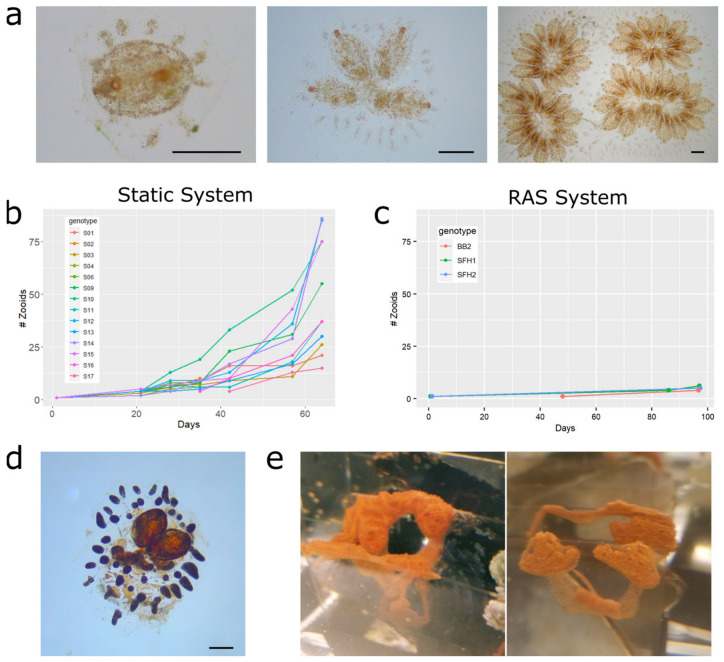
Tunicate growth in initial culture systems. Images of growth progression from a single oozooid to a multi-zooid system to a multi-system colony in a static culture system (**a**). Growth of individual *Botryllus* genotypes was quantified as the number (#) of zooids over days in culture of the static system (**b**) and the few surviving genotypes in the initial RAS (**c**). Images of regressing *Botryllus* genotype (**d**) and expanding *Botrylloides* (**e**) in initial RAS. Scale bars in each micrograph = 1 mm.

In the static system, tunicates rapidly expanded from single oozooids to multi-system colonies (Figure 2a). Least squares linear regression of ln-transformed zooid number versus time (days) resulted in the linear growth model ln(Zooids) = 0.063437 (Time) − 0.033670. This yielded an estimated growth constant of 0.063437/day (0.44/week) with an R^2^ = 0.871, corresponding to a doubling time of approximately 10.9 days (Figure 2b). In the initial RAS, some oozooids grew to a maximum of six zooids with only a couple of systems surviving up to 100 days (Figure 2c), but most would eventually regress and disappear within 60 days (Figure 2d). There were no surviving oozooids from the first BM collection beyond 100 days. Only three and two oozooids obtained from the BB and SFH collections survived beyond 100 days, respectively. In cointrast, the initial RAS was able to support *Botrylloides* spp. growth,. Easily noticeable continuous colony expansion was observed for *Botrylloides* spp., but qualitative visual observation was not substantiated with quantitative data, as *Botrylloides* spp. were not the target species (Figure 2e). Expansion of *Botrylloides* colonies continued for over a year in the initial RAS until they were culled. In contrast, no wild-caught or lab-born *B. schlosseri* survived longer than six months in the initial RAS.

### 3.4. Revised RAS

**Figure 3:**
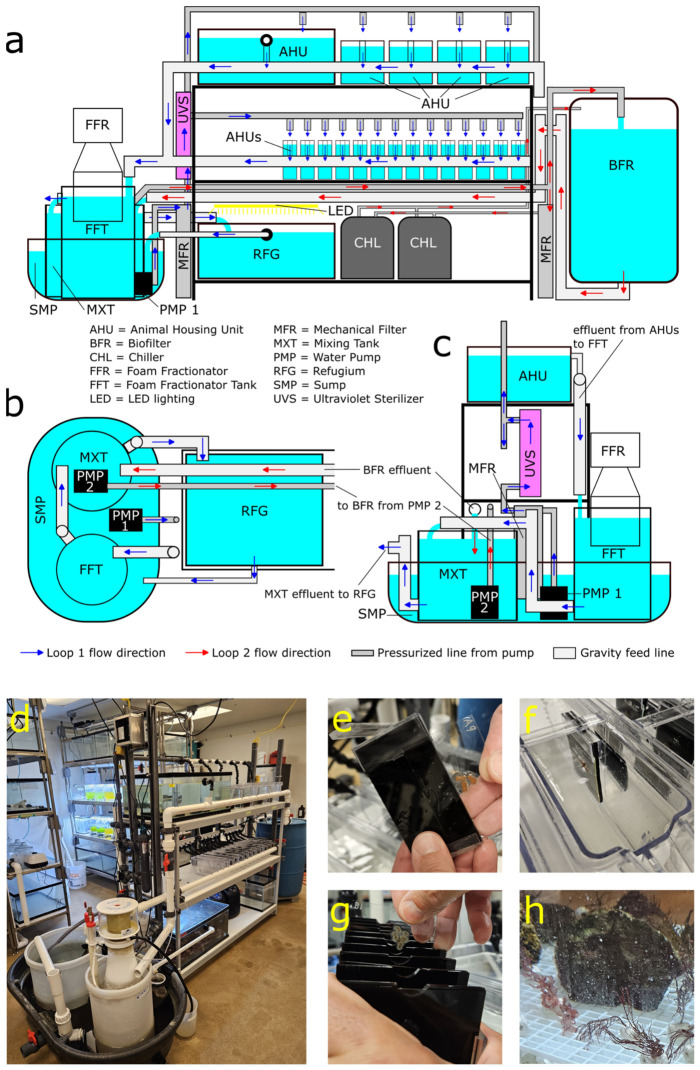
Completed revised RAS. Schematic of revised RAS with flow directionality in side view (**a**), top view of flow directionality between SMP components, RFG, and BFR (**b**), and view from SMP end (**c**). View of the entire RAS (**d**), images of black opaque-backed slide holder system, including single slide holders (**e**,**f**) and a ten-slide holder rack (**g**). Image of a mature refugium containing two actively growing macrophyte species (**h**).

Water circulation in the 760 L total volume, newly optimized, revised RAS (Figure 3a,b), is divided into two independent loops driven by separate PMPs. Loop 1 starts in the lowest elevation point of the system (SMP) with PMP1, which drives water flow at a rate of 14 L/min through an MFR, the UVS (UV exposure time of 2.65 s), then diverges into two lines feeding the AHUs on level 2 and level 3. The effluent from both levels of AHUs collects into gravity-fed lines that flow directly into the FFT containing the FFR. The FFT overflow supplies water by gravity to the MXT. The resulting MXT overflow passes through the RFG illuminated by two LEDs running at half power, before returning to the SMP. Loop 2 starts in the MXT with PMP2 driving water at a rate of 27 L/min into two lines. Line 1 flows directly into the BFR, and line 2 flows into another MFR, then through the two CHLs, and into the BFR. From here, water flows by gravity back into the MXT, where interchange between the two loops occurs. Without input from loop 1, loop 2 circles back on itself between the BFR and the MXT. If loop 2 is off, loop 1 cycles between AHUs, FFT, MXT, the RFG, and the SMP. This arrangement allows loop 1 to be turned off, preventing immediate flushing of feed from the AHUs while feeding the animals. Relative to the initial RAS, this arrangement allows maintenance of a high flow rate of loop 2 (turnover rate of 5.8 cycles/h) supporting better thermal management, increased mechanical and biological filtration efficiency, redundancy in the flexibility to operate the entire system with one loop in the case of an equipment failure, minimized flow through loop 1 (turnover rate of 2.7 cycles/h) which increases UV sterilization, longer retention time of feed in AHUs, and longer retention time of AHU effluent in FFR leading to increased efficiency of removing excess feed and deleterious tunicate waste. This new arrangement also allows animals to assimilate potential beneficial feed generated by the refugium before it is removed by the rest of the filter system. *B. schlosseri* colony-containing slides were held with either a single slide holder in 1.8 L AHUs of level 2 (Figure 3c,d) or ten slide holders in 9.5 L AHUs of level 1 (Figure 3e). At the time of the RAS remodel, all but two macrophyte varieties, *Gracilaria* sp. and *Porphyra* sp. (Figure 3f), had regressed and disappeared. These two remaining species grew rapidly and repopulated the refugium.

### 3.5. Revised RAS Growth

**Figure 4:**
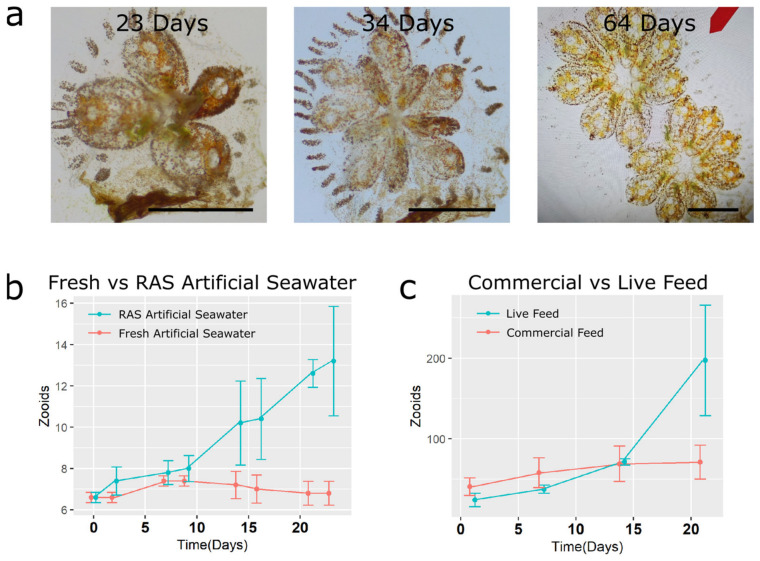
Growth of *B. schlosseri* in the optimized, revised RAS and results of optimization experiments. Images showing recovery of a surviving genotype after transfer from the initial RAS for the specified number of days to the optimized RAS (**a**). Comparisons of *B. schlosseri* growth quantified by number of zooids/time (days) in RAS water versus fresh artificial seawater (AS) (**b**) and fed either live microalgae or commercial diets (**c**). Each data point represents the mean zooid number with standard error bars for the specified time point. Sample sizes are (*n*) = 10 and (*n*) = 6 for the water and feed experiments, respectively. Scale bars in each micrograph = 2 mm.

The few remaining *B. schlosseri* genotypes from the initial RAS design immediately began to recover in the newly optimized, revised RAS. They were able to fight off biofilm contamination and parasites, and systems increased in zooid number (Figure 4a). In the optimized RAS, greater than 34 oozooids survived beyond 150 days from the first field collection after the RAS remodel (BM) with a mean RCZN of 16.5 from 18 selected genotypes in which zooid counts were recorded over that time period.

### 3.6. Superior Growth of RAS Adapted Tunicates in Static Systems When Using RAS Water

When transferring tunicates from the optimized RAS to the static system, a significant difference in growth rate was observed depending on whether they were grown using RAS water or freshly prepared AS. After a ~10-day lag period, exponential growth was observed in the RAS water-treated group, while a reduction in zooid counts was observed in the fresh AS-treated group (Figure 4b). The RAS treatment group yielded a mean RCZN of 1.95, which was significantly greater than the fresh AS group RCZN of 1.04 (*p*-value = 0.04952).

### 3.7. Elevated Growth in Response to a Live Feed (LF) Mixture over Commercial Feed

After a ~14-day lag period, an increase in colony growth rate was observed in LF-fed tunicates (Figure 4c). The LF treatment group yielded a mean RCZN of 8.37 that was significantly greater than the CF group RCZN of 1.70 (*p*-value = 0.005587).

### 3.8. Feeding Frequency Increase in the Optimized RAS

**Figure 5:**
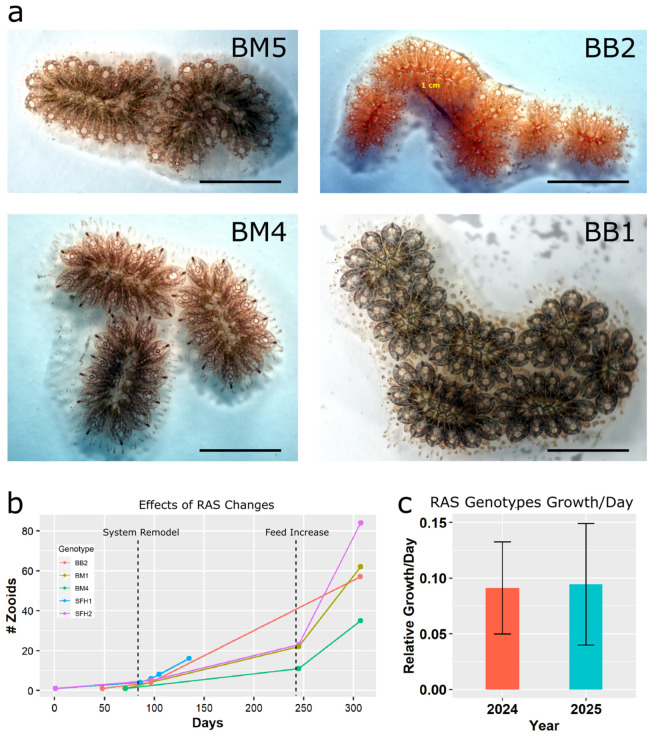
Representative images of *B. schlosseri* colonies from multiple genotypes after sustained growth in the optimized RAS (**a**). Tracking of genotype growth (those for which there were zooid count data prior to RAS remodel) over time with annotated time points of system remodel and feeding procedural changes (**b**). Growth rate (#units/day) comparison of the same genotypes in 2024 (mean = 0.091, 95% CI [0.063, 0.119]) and 2025 (mean = 0.094, 95% CI [0.058, 0.131]), showing long-term maintenance of growth rates (**c**). Scale bars in each micrograph = 5 mm.

An overall increased growth rate was apparent in multiple genotypes after increasing the feeding frequency from every other day to daily, with a rapid increase in zooid number and expansion into multi-system colonies (Figure 5a). This observation was confirmed by an additional zooid count 50 days post feeding frequency increase, resulting in steeper slopes of zooid number/day for each genotype compared to the growth rates before increasing feeding frequency in the optimized RAS (Figure 5b).

### 3.9. Long-Term Status of B. schlosseri Cultures in the Optimized RAS

At the last 2024 sampling point, 34 genotypes remained with an average zooid count of 61 zooids/genotype. Currently, 11 genotypes have been maintained and asexually propagated for over 2 years with an average of 6 slides per genotype (~6 systems/slide and ~10 zooids/system) despite regular harvesting of animals for experiments (in 2025, the number of systems per genotype was tracked to simplify record keeping). For example, 219 systems from genotype BM4, consisting of more than 6 zooids/per system, were harvested for use in a study of copper toxicity stress [24]. Growth rate (#units/day) comparison of the current genotypes between 2024 (mean = 0.091, 95% CI [0.063, 0.119]) and 2025 (mean = 0.094, 95% CI [0.058, 0.131]) shows a maintenance of consistent growth rates over time (Figure 5c). Additional signs of animal vigor in the optimized RAS include production of gametes by many genotypes with a common occurrence of sexually produced oozooids on slides. This observation demonstrates the capacity of the optimized RAS for producing mixed genotypes in a controlled manner and to sustain a laboratory population by occasional controlled sexual reproduction without additional sampling from the wild.

## 4. Discussion

The overall objective of this work was to construct a tunicate culture system capable of sustaining and propagating healthy *Botryllus schlosseri* lab strains at a rate sufficient to support extensive research using this model organism without reliance on natural seawater. The static system resulting from these efforts was successful at achieving this goal and is well-suited to isolate specific experimental contexts. But with a weekly 100 percent water turnover and cleaning of every AHU, the static system becomes less practical for long-term culture and propagation with increasing scale. An additional objective was to develop a higher capacity RAS to: 1. reduce maintenance and cost of consumable resources; 2. create a more natural, biologically relevant culture environment; and 3. advance ecologically sustainable recirculating aquaculture system (RAS) techniques for these animals. An artificial culture system must balance replication of specific required environmental parameters, practical application and feasibility, and sufficient production output of animals to meet the goals of producing healthy animals for research at a proper scale. The initial RAS version contained all the typical components of a closed-loop marine culture system and maintained basic water quality parameters within acceptable limits for the culture of many marine invertebrates. Despite the apparent proper function of this initial RAS version, no *B. schlosseri* systems were able to grow larger than 6 zooids before completely regressing. This observation was in stark contrast to the exponential growth observed in the static system at this time.

### 4.1. Potential Factors Influencing B. schlosseri Growth in Closed Loop System Cultures

Many factors can potentially influence tunicate growth, including abiotic physical parameters (temperature, salinity, lighting), density, food quality and availability, the presence or absence of inhibitory biofilms, pathogens, and interaction with allelopathic compounds. The abiotic parameters of the RAS were consistent with those in the static system and standard conditions from the literature and were thus assumed not to be the primary reason for the differences in *B. schlosseri* growth. Intriguingly, in contrast to *B. schlosseri*, *Botrylloides* spp. grew steadily in the initial RAS version. Therefore, feed quantity was also not considered a primary factor for the lack of sustained *B. schlosseri* growth. Rather, the presence of one or more other limiting factors specific to the interaction between *B. schlosseri* and the RAS initial version must be considered to explain the lack of sustained growth. In a closed-loop system like RAS, there is potential for the accumulation of specific compounds that inhibit the growth of aquatic animals [25,26,27,28] if not effectively removed by the water treatment system. The weekly 100% water change in the static system would prevent any accumulation and consequences from long-term retention of these wastes and harmful byproducts. With respect to many solutes, marine species are typically adapted to clean environments and thus have low tolerance to toxins such as nitrogenous waste [29,30]. In an artificial system, a clean environment is relatively easy to achieve with low animal densities and only maintenance-level feed. However, the goal of a production-focused system is to achieve high animal densities and rapid growth, which requires an abundant nutrition source and results in elevated waste. In a closed loop system, this can accelerate accumulation of inhibitory compounds, including metabolic byproducts (such as nitrogenous waste) and allelopathic toxins from target species, other species (*Botrylloides* spp.), and microbial decomposition of organics.

Nevertheless, many marine invertebrates, such as corals, can be proficiently cultured in RASs. Corals, through their symbiont photosynthetic microbes, have the potential to simultaneously reduce potentially toxic nitrogenous waste in the ambient environment and convert them to nutrients for supporting growth [31]. Along with decomposing bacteria, they can assimilate and convert inorganic nitrogen back to usable nutrients [32]. In this regard, corals would have an advantage over purely heterotrophic filter feeders (such as bivalve mollusks and tunicates) in closed-loop recirculating systems, which would require a higher density of suspended food to achieve rapid growth rates at the expense of water quality. Bivalves have been successfully cultured in RAS [19], but these animals have been shown to have higher tolerance to water quality parameters such as nitrogenous waste than other marine invertebrates [33]. From this study and others, it is evident that at least some *Botrylloides* species are also amenable to RAS culture [21]. It is possible that *B. schlosseri* has a higher sensitivity to water quality than *Botrylloides* spp., rendering it more susceptible to nitrogenous waste toxicity below the detection limits of the methods used in this study or to some other metabolic waste byproduct. Excess waste can also negatively impact water quality indirectly through its influence on microbial communities. Excess inorganic nutrients and organics can support photosynthetic (i.e., cyanobacteria) and heterotrophic (i.e., *Vibrio* spp.) harmful microbes, respectively, both of which are known to release exotoxins that are highly toxic to aquatic animals [34,35].

Allelopathic compounds generated by the tunicates themselves represent another potential factor for limiting *B. schlosseri* growth. The ability to inhibit the establishment of nearby competitors of sessile marine invertebrates is a common phenomenon seen in sponges [36], corals [37] and anemones [38]. Many reports support the notion of a soluble factor released by colonial tunicates capable of suppressing the growth of other tunicate species or conspecific genotypes [39,40]. Growth of *Botrylloides leachii* was observed to be roughly half in the proximity of other sessile invertebrates without physical contact, relative to *B. leachii* grown in isolation [41]. These data indicate the presence of inhibitory soluble factors derived from competing organisms. Some of these ascidian-derived allelopathic compounds have been identified [42]. Multiple ascidian secreted alkaloids have demonstrated suppressive effects on general eukaryotic cell viability and proliferation [43,44,45]. It is possible that such competitive inhibitory factors are released by the tunicates and not efficiently removed by the filter system and, thus, accumulate in the circulating water. If such soluble compounds possess potent enough activity to have biological effects in an environment as dilute as the ocean, it is conceivable they could accumulate to impactful concentrations in a recirculating system.

### 4.2. RAS Optimization

Similar observations of prolific *Botrylloides* growth but minimal Botryllus growth were made between our initial RAS and another report of RAS-cultured colonial tunicates [21]. In both systems, the foam fractionator is downstream of the refugium in the sump, where mixing with multiple inputs from the system occurs.

In an attempt to account for the potential deleterious factors discussed above and improve the RAS of *B. schlosseri*, the system was remodeled with foam fractionation confined to an isolated chamber that received effluent directly from the AHUs, from which the effluent flows to the biological filtration loop, followed by the refugium.

This optimized arrangement was designed to achieve three key goals: to maximize removal of excess feed and deleterious tunicate byproducts by increasing AHU effluent retention time in the FF; to prevent mixing and redistribution of AHU waste effluent before passing through the complete water treatment cycle; and to minimize removal of potentially beneficial refugium products before distribution to the tunicates. Foam fractionation water treatment has many benefits [46] including: removal of suspended solids, dissolved organic carbon and a variety of biochemically/pharmaceutically active compounds [47,48,49]. It also improves the biological filter nitrification [50].

Macrophytes release substantial dissolved and particulate organic matter into the ambient water [51,52]. This potential nutritional supplement for filter-feeding tunicates also includes allelopathic compounds that inhibit biofouling by microorganisms [53,54]. Since they often live in close association with macroalgae, this could benefit *B. schlosseri*, as tunicates can be strongly inhibited by biofilms [55], which may partially explain why laboratory-cultured *B. schlosseri* typically require manual cleaning to assist in fighting biofilms relative to the wild, where they thrive amidst a complex biome.

Additionally, inorganic nutrients are deleterious to marine invertebrate physiology even at low sublethal levels [29,56,57,58] can be reduced by macroalgae direct assimilation [59] and improvement of BF nitrification performance [60]. Over the multiple years of this work, qualitative observation suggests a correlation between refugium and tunicate health. During periods of decaying organic matter accumulation from dying macrophytes, tunicate growth stalled. In contrast, when the refugium was stable with positive macrophyte growth from the remaining well-adapted species, improved tunicate condition and growth followed. However, this observation is anecdotal, requiring additional quantitative experiments to determine if this apparent relationship is correlative or causative.

The establishment of a stable refugium was accompanied by dividing the flow between two pumps, one driving flow to the AHUs and another driving flow through a mechanical filter, the temperature control, and the biofilter. This arrangement allowed minimizing flow to the AHUs to increase retention time with the feed after feeding while maintaining high flows through the rest of the filtration system. Another notable change that contributed to the success of the optimized RAS version was holding tunicate containing glass slides in slide holders having a black, opaque backing. This change was aimed at creating a more natural substrate for tunicate growth by preventing light exposure to the back of the slides and, thus, reducing competition with unwanted growth of photosynthetic microbes underneath the tunic.

### 4.3. Feed

The optimized fluidics of the RAS, the maturation of the refugium, and the removal of competitor *Botrylloides* spp. from the system all contributed to overall improved water quality and promoted long-term survival, better condition, and moderate growth rates of *B. schlosseri*. However, the results from the static system clearly indicated that much higher growth rates were possible. A challenge of a production-focused RAS for a more sensitive species is the maintenance of sufficient water quality despite high organic loads [61]. Thus, it is critical to discern the ideal compromise between the cleanliness of the water and feed availability for the target species. Feeding was initially minimized due to concerns of fouling the system with excess feed. However, comparison of optimized RAS water with fresh AS from the static system indicated that the RAS water was as supportive of *B. schlosseri* growth as AS, and the differences in growth rates between the two systems could be due to the flow regimen causing shorter feed retention in RAS compared to the static system.

Retention time with the feed and thus nutrient availability is a clear advantage of the static system, and thus it was concluded that once whatever inhibitory factors were removed at the time of system remodel, then feed availability became the new limiting factor and the primary cause of the differences in growth rates observed between the static and revised RAS. This rationalization inspired the change in the RAS feeding frequency from every other day to daily. Improved efficiency of excess feed removal in the remodeled RAS allowed for this increase without compromising water quality.

Commercial feed was used in the initial stages of this project due to its immediate accessibility and ease of application, which was sufficient to achieve growth in the static system. When unsatisfactory results were obtained using these products in the RAS, live feed was considered as a potential option for improvement, as the performance of many cultured filter-feeding animals is improved using live feeds [62]. The feed comparison experiment indicated that a live feed combination supported superior growth rates of *B. schlosseri* when compared to the commercial concentrate tested. An additional advantage of live feeds is their motility and resistance to decay [62], in contrast to non-living commercial feed, which can lead to non-beneficial microbial growth and contamination of the tunic. These attributes are especially important in long-term, continuous RAS culture, where the build-up of non-living organic matter can have negative consequences on water quality [63].

### 4.4. Limitations and Future Directions

Many of the key aspects influencing *B. schlosseri* culture were addressed during the course of this study, and proficient propagation of tunicates has been achieved using both static and RAS approaches. The main and ultimately most meaningful parameter of system performance was the growth and reproduction of the species of interest. However, as total tunicate biomass increases and for further scaling up the capacity of RAS, it is possible that the previous limiting factors could overwhelm the current system, requiring additional system improvements to sustain the same growth rates. Further optimization of the current system components is one approach to improve performance even more. A limitation is that many of the design attributes were not implemented in isolation, and thus, the relative contribution of each individual component is currently unknown. Future analyses that quantify the contribution of biochemical and biological water quality parameters to system performance would be beneficial by identifying where further optimizations can be made. Possible future improvements include: heterotrophic plate counts measuring bacteria load and UV sterilizer proficiency in reducing microbes; dissolved organic carbon (DOC) testing at different points of the circulation loop to evaluate different configurations of foam fractionation; more sensitive, quantitative nitrogenous waste testing (i.e., spectrophotometric methods); and tunicate growth trials in the presence or absence of different macrophytes. Incorporating new system components is another approach to increasing capacity. As a powerful oxidant, adding ozone (O_3_) to the FFT could inactive biochemical inhibitors, reduce the bioavailability of dissolved organics, and sterilize nuisance microorganisms. Adding ozone (O_3_) to the FFT is an option that could increase the carrying capacity of the RAS even further. This would be especially beneficial in the case of organic allelopathic compounds discussed earlier, which may be difficult to remove by other treatment methods. As the system is currently constructed, there is significant residence time in the treatment loop to dissipate O_3_ and UV exposure to revert residual O_3_ back to O_2_ before treated water returns to the animals. Additionally, the results from this work indicate animal retention time with the feed as a critical determinant of *B. schlosseri* growth and, thus, development of an automatic feeding system coupled with intermittently paused flow is worth considering if increased production capacity is needed.

## 5. Conclusions

The culture systems described here have been demonstrated to be capable of long-term culture of healthy *B. schlosseri* for at least several years, each with specific strengths that can be used in combination to support research using this model organism. The static method requires more maintenance per animal/AHU but allows for isolation of animals into distinct units in which different experimental treatments can be applied. The RAS does not allow for complete isolation of animal groups (e.g., different water quality and temperature) but can proficiently maintain and propagate separate genotype lines of *B. schlosseri* and serve as a source of laboratory strain animals for experiments. This workflow has several advantages over natural seawater-sourced systems. As reproducible artificial systems, comparability of results is increased by eliminating variability associated with the source water, which can vary seasonally and stochastically. Other important advantages are increased biosecurity and containment of transgenic animals without the risk of genetically modified material escaping into the effluent to the environment. The cumulative modifications introduced during optimization of the RAS and associated husbandry protocols guided by careful consideration of the most plausible influential factors have led to a long-term production capability of healthy animals sufficient to support extensive research efforts for over two years to date. Therefore, the systems documented here represent valuable resources for the scientific community to propel research using these intriguing tunicate models.

## Data Availability

Data will be made available on request.

## Abbreviations

The following abbreviations are used in this manuscript:

RAS	Recirculating aquaculture system
BM	Berkeley Marina
BB	Bodega Bay
SFH	San Francisco Harbor
AHU	Animal housing unit
RFG	Refugium
SMP	Sump
PMP	Pump
BFR	Biofilter
MFR	Mechanical filter
FFR	Foam fractionator
FFT	Foam fractionator tank
UVS	Ultraviolet sterilizer
MXT	Mixing tank
CF	Commercial feed
LF	Live feed
RCZN	Relative change in zooid number
